# The Mediating Effects of Attachment Styles on the Relationship between Sensory Processing Styles and Interpersonal Problems in Healthy University Students

**DOI:** 10.1155/2020/6204120

**Published:** 2020-05-06

**Authors:** Oan Na Lee, Gyeong-A Park

**Affiliations:** Department of Occupational Therapy, Chosun University, Gwangju, Republic of Korea

## Abstract

**Purpose:**

Difficulties with sensory processing are known to have negative effects on individuals' attachment styles and the interpersonal domain. We investigated the relationships among sensory processing styles, attachment styles, and interpersonal problems to better understand the role of attachment styles on the relationship between sensory processing styles and interpersonal problems. *Participants.* One-hundred and eighty-four university students (aged 18-28 years) completed a set of self-reported measures.

**Methods:**

Sensory processing styles, attachment styles, and interpersonal problems were assessed with the Adolescent/Adult Sensory Profile, Experiences in Close Relationships-Revised, and Inventory of Interpersonal Problems-Short Circumplex Form, respectively.

**Results:**

Low registration (*r* = 0.587, *p* < 0.001) and sensory avoidance (*r* = 0.501, *p* < 0.001) were positively correlated with interpersonal problems. Regression analyses indicated that low registration (*β* = 0.301, *p* < 0.001) and anxious attachment (*β* = 0.640, *p* < 0.001) were predictors of interpersonal problems (*R*2 = 0.672, *p* < 0.001), and sensation avoidance (*β* = 0.386, *p* < 0.001) and avoidant attachment (*β* = 0.233, *p* < 0.001) were predictors of interpersonal problems (*R*2 = 0.286, *p*<0.001). Participants with higher levels of low registration reported higher levels of interpersonal problems, and this relationship was partially mediated by anxious attachment. Participants with higher levels of sensory avoidance reported higher levels of interpersonal problems, and this relationship was partially mediated by avoidant attachment.

**Conclusions:**

This study clarifies the relationships between sensory processing styles and interpersonal problems and the mediating effects of attachment styles. The results were discussed in light of the related literature.

## 1. Introduction

Sensory processing is the individuals' ability to manage incoming sensory stimulation, and it relates to the way the individual recognizes, modulates, perceives, and responds to sensory stimulation [1]. Dunn [2] categorized the characteristics of sensory processing into four styles based on the results of the interaction between the neurological threshold continuum and behavioral response continuum. Low registration is characterized by passively responding to a high neurological threshold, recognize and respond to an external stimuli slowly, and not actively seeking sensory stimulation. Individuals showing the characteristics of low registration can experience difficulty recognizing or expressing their own internal emotional state or inferring the emotions of others through their behaviors. Furthermore, they may not initiate relationships. Sensory seeking is characterized by actively responding to a high neurological threshold. Individuals showing the characteristics of sensory seeking seek strong stimulation, enjoy stimulating environments and activities, and sometimes display risky behaviors. Sensory sensitivity is characterized by passively responding to a low neurological threshold. Individuals showing the characteristics of sensory sensitivity experience discomfort with sensory information and are easily overwhelmed by it, but do not actively limit their exposure to uncomfortable stimulations. Finally, sensory avoidance is characterized by actively responding to a low neurological threshold. Individuals showing the characteristics of sensory avoidance actively limit being exposed to strong sensory stimulation. If problems occur with sensory processing and individuals cannot appropriately respond to and process external sensory stimulation, they can experience difficulties with daily life by responding sensitively or becoming dull to sensory stimulation and display dysfunctional behaviors [3]. These dysfunctional sensory processing patterns can have a negative impact on not only functional performance in an individual's daily life [4, 5] but also on social relationships [6] and social skills [7].

Sensory processing affects an individual's daily life activities such as social participation and social skills [8–10]. Specifically, individuals with sensory avoidance and sensory sensitivity styles may experience tension and anxiety, and they can show maladaptive behavioral tendencies in the relationships with others [6, 11–13]. According to the results of these previous studies, it can be predicted that the style of sensory processing of an individual is a personal internal/trait factor that affects interpersonal relationships. Another aspect of life that sensory processing can have effect on is individuals' attachment styles [6]. Attachment refers to the emotional bond one feels with someone close [14]. The attachment formed through interactions with the caregiver during childhood affects the interpersonal relationships—especially close relationships—in adulthood [15]. Therefore, attachment theory provides a framework in which the interpersonal relationship problems of an individual can be viewed [16].

Fraley and Waller [17] examined the characteristics of adult attachment: anxious attachment and avoidant attachment. Individuals with anxious attachment have a tendency for an excessive desire for the attention and affection of others and fear of abandonment from others. Individuals with avoidant attachment have a tendency of excessive self-reliance and fear of becoming close with others. Therefore, individuals with high anxious attachment excessively obsess over or depend on the close figure due to the fear of being rejected or abandoned [18]. Individuals with high avoidant attachment attempt to maintain their independence from others [18, 19]; they try hard to avoid a relationship rather than suffer anxious from the conflicts and difficulties a relationship entails [19]. In a systematic review by Hayden and colleagues [20], strong associations between adult attachment styles and interpersonal problems were found. Thus, it is clear that people with high anxious attachment or avoidant attachment experience psychological difficulties and difficulties with interpersonal relationships despite the different appearances of the two styles.

Empirical studies have reported a relationship between sensory processing styles and attachment styles. Specifically, low registration was found to have a positive correlation with anxious attachment and avoidant attachment [6]. Furthermore, the sensory sensitivity style was found to have a positive relationship with anxious attachment [21] and avoidant attachment [6], and the sensory avoidance also was found to have a positive correlation with anxious attachment [21] and avoidant attachment [6, 21]. However, the sensory seeking style was found to have a negative relationship with avoidance attachment and no correlation with anxious attachment [21]. Individuals with low registration, sensory sensitivity, and sensory avoidance processing styles may experience tension and anxiety by passively responding, actively avoiding, or strongly seeking out external stimulation and can show maladaptive behavioral tendencies in response to the formation of relationships with others and toward the environment. Individuals with a sensory seeking processing style actively seek stimulation and thus may experience neither tension nor anxiety with others.

Considering the effect of sensory processing on attachment styles and interpersonal relationship, and the effect of attachment styles on interpersonal relationship, it is logical to predict that the attachment styles mediate the relationship between the sensory processing styles and the interpersonal problems. Although the previous studies indicate that the sensory processing patterns and attachment styles can have impacts on individuals' daily lives [4, 5], social relationships [6, 19, 20], and social skills [7], there is no study examining the relationships among sensory processing styles, attachment styles, and interpersonal problems in healthy individuals. Since individuals' sensory processing styles and attachment styles are found to have effects on daily lives and social relationships, it is important to examine the role of the sensory processing patterns and attachment styles on the interpersonal problems of university students who are in a difficult period due to difficulties in identity development and interpersonal relations [22]. Thus, the present study aimed to explore the mediating effects of attachment styles on the relationship between sensory processing styles and interpersonal problems and to identify the cause of interpersonal problems to plan interventions.

## 2. Materials and Methods

### 2.1. Procedures

The research was approved by the Institutional Review Board of Chosun University (2-1041055-AB-N-01-2018-52). Participants were recruited by flyers posted at universities in Gwangju and Jeollanamdo, South Korea. After providing a written informed consent, participants completed questionnaires consisting of a demographic questionnaire, the Adolescent/Adult Sensory Profile, Experiences in Close Relationships-Revised Questionnaire, and Inventory of Interpersonal Problems-Short Circumplex Form.

### 2.2. Participants

Self-reported surveys were obtained from 184 university students in Gwangju and Jeollanamdo, South Korea. Participants consisted of 84 (45.7%) males and 100 (54.3%) females with a mean age of 20.9 years (SD = 2.0). Among them, 51 (27.7%) were first-year, 55 (29.9%) were second-year, 44 (23.9%) were third-year, and 34 (18.5%) were fourth-year undergraduate students.

### 2.3. Measures

#### 2.3.1. Adolescent/Adult Sensory Profile

The Korean version of the Adolescent/Adult Sensory Profile (K-AASP; [23]) that was validated in Korean is a 60-item self-report scale that was designed to assess individuals' responses to sensory experiences [24]. AASP was translated into Korean to take into account the social and cultural context of Korea and back-translated into English by bilingual occupational therapists [23]. Sixty items are sorted among four quadrants for scoring: low registration (15 items), sensation seeking (15 items), sensation sensitivity (15 items), and sensation avoidance (15 items). Each item is scored on a 5-point Likert scale ranging from 1 (almost never) to 5 (almost always) and the score range for each quadrant is from 15 to 75. Higher scores indicate that individuals experience greater difficulties with the respective sensory processing. Based on Korean samples of 1,192 adolescents and adults (aged 11-90 years), norms were defined for adolescents (aged 11-18 years), adults (aged 19-64 years), and older adults (aged 65 and up). Normal ranges for adults (aged 18-64) are as follows: 22 to 34 for low registration, 30 to 43 for sensation seeking, 27 to 40 for sensation sensitivity, and 27 to 40 for sensation avoidance. The Korean version of AASP has good internal consistency, with coefficient alpha values for low registration, sensation seeking, sensation sensitivity, and sensation avoidance are .785, .705, .750, and .771, respectively. In the current study, coefficient alpha values for low registration, sensation seeking, sensation sensitivity, and sensation avoidance are.817, .738, .747, and.798, respectively. Since coefficient alpha values are higher than.7, the internal consistency which assesses whether items in the scale measure the same construct is considered to be acceptable.

#### 2.3.2. Experiences in Close Relationships-Revised Questionnaire

The Korean version of the Experiences in Close Relationships-Revised Questionnaire (ECRR-K; [25]) is a 36-item self-report scale designed to measure romantic attachment style [26]. This questionnaire was validated in Korean version: It was translated into Korean to take into account the social and cultural context of Korea and back-translated into English by bilingual psychologists [25]. It consists of two 18-item scales: anxious attachment and avoidant attachment. Each item is scored on a 7-point Likert scale ranging from 1 (strongly disagree) to 7 (strongly agree), and the score range for each attachment style is from 18 to 126. ECRR-K does not provide normal ranges. However, higher scores indicate greater relationship insecurity. The Korean version of ERR-R had good internal consistency, with coefficient alpha values for anxious attachment and avoidant attachment are .89 and .85, respectively. In the current study, coefficient alpha values for anxious attachment and avoidant attachment are.944 and.833, respectively. Since coefficient alpha values are higher than.7, the internal consistency which assesses whether items in the scale measure the same construct is considered to be acceptable.

#### 2.3.3. Inventory of Interpersonal Problems-Short Circumplex Form

The short form of the Korean Inventory of Interpersonal Problems Circumplex Scale (KIIP-SC; [27]) is a 40-item self-report scale designed to measure the level of interpersonal problems that individuals experience [27]. This questionnaire was validated in Korean version: It was translated into Korean to take into account the social and cultural context of Korea and back-translated into English by bilingual psychologists [27]. Each item is scored on a 5-point Likert scale ranging from 1 (strongly disagree) to 5 (strongly agree), and the score range of the inventory is from 40 to 200. KIIP-SC does not provide normal ranges. However, higher scores indicate greater interpersonal problems. The Korean version of IIP-SC had good internal consistency, with a coefficient alpha value of.89. In the current study, coefficient alpha value is.948. Since a coefficient alpha value is higher than.7, the internal consistency which assesses whether items in the scale measure the same construct is considered to be acceptable.

### 2.4. Data Analysis

To determine the relationships among sensory processing styles, attachment styles, and interpersonal problems, descriptive statistics, Pearson correlation analysis, and regression analyses were analyzed by using SPSS 24.0 for Windows. To examine the mediating effects of attachment styles on the link between sensory processing styles and interpersonal problems, Baron and Kenny's [28] recommendations for mediation analysis were followed. First, a significant prediction of the independent variable to the mediator is required. Second, a significant prediction of the independent variable to the dependent variable is required. Third, when the independent and the mediator concurrently predict the dependent variable, a previously significant prediction of the independent variable to the dependent variable is no longer significant or decreases. Fourth, if the significant prediction of the independent variable to the dependent variable is no longer significant, it indicates that the mediator fully mediates the relationship between the independent variable and dependent variable. If a significant prediction of the independent variable to the dependent variable decreases, it indicates that the mediator partially mediates the relationship between the independent variable and dependent variable. In this study, the independent variable, the mediator, and the dependent variable were four sensory processing styles, attachment styles, and interpersonal problems, respectively. Statistical significance was set at *p* < 0.05.

## 3. Results

### 3.1. Descriptive Statistics of Variables

The means and standard deviations of low registration, sensation seeking, sensation sensitivity, and sensation avoidances were 33.4 ± 7.3, 38.4 ± 7.9, 34.5 ± 8.0, and 39.4 ± 7.9, respectively. These means were within a normal range. However, the means of low registration and sensation avoidance were located in the upper end of normal ranges. The means and standard deviations of anxious attachment and avoidant attachment were 42.9 ± 14.4 and 47.5 ± 10.4, respectively. The mean and the standard deviation of interpersonal problems was 89.2 ± 23.9.

### 3.2. Correlations among Variables

The correlations of variables are presented in Table 1. There were significant correlations between sensory processing styles and attachment styles except for sensation seeking and anxious attachment. There were also positive correlations between sensory processing styles and interpersonal problems, except for sensation seeking and interpersonal problems.

### 3.3. Effects of Sensory Processing Styles on Attachment Styles and Interpersonal Problems

Three multiple linear regression analyses were applied to examine the effects of sensory processing styles on attachment styles and interpersonal problems (Table 2). It was found that low registration (*β* = .240, *p* < 0.01) and sensation sensitivity (*β* = .232, *p* < 0.05) were significant predictors of anxious attachment (*R*2 = .262, *p* < 0.001). Sensation seeking (*β* = −.316, *p* < 0.001) and sensation avoidance (*β* = .334, *p* < 0.001) were significant predictors of avoidant attachment (*R*2 = .295, *p* < 0.001). Low registration (*β* = 0.454, *p* < 0.001) and sensation avoidance (*β* = .272, *p* < 0.01) were significant predictors of interpersonal problems (*R*2 = .407, *p* < 0.001).

### 3.4. Mediation of Anxious Attachment on Relationship between Low Registration and Interpersonal Problems

**Figure 1:**
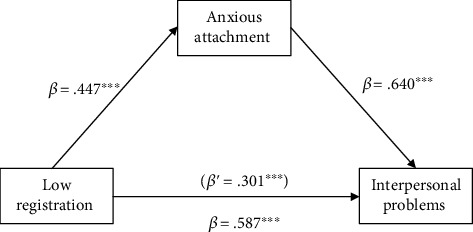
Results of testing for mediation by anxious attachment between low registration and interpersonal problems. Note: ∗∗∗*p* < 0.001.

The four-step model outlined by Baron and Kenny [28] was followed to examine whether the anxious attachment was a mediating variable that accounts for the relationship between low registration and interpersonal problems. The first model tested the direct effect of low registration on anxious attachment, yielding a significant result (*β* = .447, *p* < 0.001). Also, a significant direct effect of low registration on interpersonal problems was shown (*β* = .587, *p* < 0.001). Next, both low registration and anxious attachment were entered into a regression equation. The standardized coefficients (*β*) for low registration (*β* = .301, *p* < 0.001) and anxious attachment (*β* = .640, *p* < 0.001) remained significant (*R*2 = .672, *p* < 0.001). The standardized coefficient for low registration was decreased. Thus, the relationship between low registration and interpersonal problems was partially mediated by anxious attachment (Figure 1). According to Baron and Kenny [28], mediating effects may present if an independent variable has a significant effect on a mediator and dependent variable. Therefore, the mediating effects of anxious attachment on the relationships between other sensory processing styles and interpersonal problems were not applied since sensation seeking, sensation sensitivity, and sensation avoidance were not predictors of either anxious attachment or interpersonal problems (Table 2).

### 3.5. Mediation of Avoidant Attachment on Relationship between Sensation Avoidance and Interpersonal Problems

**Figure 2:**
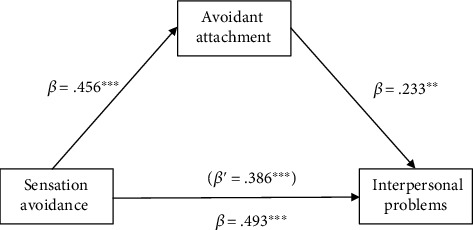
Results of testing for mediation by avoidant attachment between sensation avoidance and interpersonal problems. Note: ∗∗*p* < 0.01, ∗∗∗*p* < 0.001.

The four-step model outlined by Baron and Kenny [28] was followed to examine whether the avoidant attachment was a mediating variable that accounts for the relationship between sensation avoidance and interpersonal problems. The first model tested the direct effect of sensation avoidance on avoidant attachment (*β* = .456, *p* < 0.001). Also, a significant direct effect of sensation avoidance on interpersonal problems was found (*β* = .493, *p* < 0.001). Next, both sensation avoidance and avoidant attachment were entered into a regression equation. The standardized coefficients (*β*) for sensation avoidance (*β* = .386, *p* < 0.001) and avoidant attachment (*β* = .233, *p* < 0.001) remained significant (*R*2 = .286, *p* < 0.001). The standardized coefficient for sensation avoidance was decreased. Thus, the relationship between sensation avoidance and interpersonal problems was partially mediated by avoidant attachment (Figure 2). According to Baron and Kenny [28], mediating effects may present if an independent variable has a significant effect on a mediator and dependent variable. Therefore, the mediating effects of avoidant attachment on the relationships between other sensory processing styles and interpersonal problems were not applied since low registration, sensation seeking, and sensation sensitivity were not predictors of either avoidant attachment or interpersonal problems (Table 2).

## 4. Discussion and Conclusion

The aim of this study was to investigate the relationships between sensory processing styles and interpersonal problems, and the mediating effects of attachment styles between them. The results indicate that anxious attachment served as a mediator between low registration and interpersonal problems, indicating that adults with low registration reported interpersonal problems if low registration was accompanied by greater anxious attachment. Also, avoidant attachment served as a mediator between avoiding sensation and interpersonal problems, indicating that adults with avoiding sensation reported interpersonal problems if avoiding sensation was accompanied by greater avoidant attachment.

We also examined the relationship between sensory processing styles and interpersonal problems and found that only low registration and sensory avoidance had impacts on interpersonal problems. It appears that individuals with a high tendency of low registration are less responsive respond to external stimulation and have difficulty with interpersonal relationships [10, 29–31]. Also, individuals with a high tendency of sensory avoidance experience tension and anxious, maladaptive behavioral tendencies toward external stimulation and others, resulting in difficulties with interpersonal relationships [6, 11–13, 29–31]. These results are in line with the results of previous studies that individuals with a higher tendency of sensory avoidance and low registration tend to display dysfunctional behavior, and it may have a negative effect on their social skills and they may experience difficulty with interpersonal relationships [6, 7, 29, 30].

As anticipated, the finding showed that anxious attachment is a mediator in the relationship between low registration and interpersonal problems. The result of this study is consistent with previous studies. According to previous studies, low registration was found to have a positive correlation with anxious attachment [6], and anxious attachment affects the interpersonal problems [20]. The results of the current study indicate that individuals who passively responding to sensory stimulation may not display an appropriate response and feel anxious, and form an anxious attachment with others [6, 30] which may lead to difficulties with interpersonal relationships [20]. In addition, the finding showed that avoidant attachment is a mediator in the relationship between sensory avoidance and interpersonal problems. The result of this study is consistent with previous studies. According to previous studies, avoiding sensory stimulation was found to have a positive correlation with avoidant attachment [6], and avoidant attachment affects the interpersonal problems [20]. The results of the current study indicate that individuals who avoid sensory stimulation may avoid stimulation from others, show maladaptive behavioral tendencies in the formation of relationships with others [11, 30], and form an avoidant attachment with others which may lead to difficulties with interpersonal relationships [20]. Thus, individuals with a high tendency of low registration and sensory avoidance may experience interpersonal problems if they form anxious attachment and avoidant attachment with others, respectively.

The findings of this study highlight the role of attachment styles in the relationship between sensory processing styles and interpersonal problems. It is possible that intervene either one of sensory processing or attachment styles may improve the interpersonal relationship. These findings have some implications for the practice of occupational therapy. Once a person has sensory processing difficulties, clinicians could directly consider their effects on the interpersonal problems. Moreover, clinicians also pay attention to the person's attachment style, because we have known that sensory processing difficulties also indirectly affect interpersonal problems from the attachment style. Clinicians may thus be more deeply and clearly identify the cause of interpersonal problems and thus planning more precise interventions.

The limitation of this study is as follows. The subjects of this study are limited to university students in the Gwangju and Jeollanamdo region; it is difficult to generalize the results of this study, and therefore caution must be taken when interpreting the results of this study. Therefore, it may be necessary to conduct a replication study involving a more diverse group of research subjects.

To our knowledge, this study is the first to examine the relationship between sensory processing styles and interpersonal problems, and the mediation effects of attachment styles on the relationship between them. The present study contributes to our understanding of the effects of sensory processing styles in everyday interpersonal life, and the role of attachment styles in the relationship between sensory processing styles and interpersonal problems. Based on the findings of the current study, an intervention on insecure attachment for individuals who experience difficulties with sensory processing and interpersonal problems may improve the quality of their social activities and interpersonal relationships.

## Figures and Tables

**Table 1:** Intercorrelations of sensory processing, attachment styles, and interpersonal problems.

Variables	1	2	3	4	5	6	7
1. Low registration	1						
2. Sensation seeking	.274∗∗∗	1					
3. Sensation sensitivity	.610∗∗∗	.119	1				
4. Sensation avoidance	.479∗∗∗	-.082	.689∗∗∗	1			
5. Anxious attachment	.447∗∗∗	.128	.460∗∗∗	.382∗∗∗	1		
6. Avoidant attachment	.226∗∗	-.305∗∗∗	.323∗∗∗	.457∗∗∗	.388∗∗∗	1	
7. Interpersonal problems	.587∗∗∗	.081	.476∗∗∗	.501∗∗∗	.774∗∗∗	.410∗∗∗	1

∗∗*p* < 0.01, and ∗∗∗*p* < 0.001.

**Table 2:** Multiple regressions of sensory processing on attachment styles and interpersonal problems.

Variables	Anxious attachment	Avoidant attachment	Interpersonal problems
Low registration	.240∗∗	.115	.454∗∗∗
Sensation seeking	.044	-.316∗∗∗	-.022
Sensation sensitivity	.232∗	.061	.014
Sensation avoidance	.111	.334∗∗∗	.272∗∗
R^2^	.262∗∗∗	.295∗∗∗	.407∗∗∗

∗*p* < 0.05, ∗∗*p* < 0.01, and ∗∗∗*p* < 0.001.

## Data Availability

Data used to support the findings of this study are available from the corresponding author.
